# Cardiac safety of L-Annamycin: a pooled analysis of five clinical trials

**DOI:** 10.3389/fcvm.2026.1817847

**Published:** 2026-09-03

**Authors:** Ziad Zalaquett, Stuart Waymack, Patrick Collier

**Affiliations:** 1 Department of Cardiovascular Medicine, Heart, Vascular, and Thoracic Institute, Cleveland Clinic, Cleveland, OH, United States; 2 Department of Statistics, Virginia Tech, Blacksburg, VA, United States

**Keywords:** acute myelogenous leukemia, annamycin, anthracycline, cardiotoxicity, soft tissue sarcoma

## Abstract

**Background:**

Anthracyclines are highly effective chemotherapeutic agents but are limited by cumulative, dose-dependent cardiotoxicity, leading to strict lifetime exposure thresholds. L-Annamycin is a next-generation liposomal anthracycline designed to preserve antitumor efficacy while reducing cardiotoxicity.

**Methods:**

We performed a pooled analysis of cardiac safety data from five sponsor- and investigator-initiated clinical trials evaluating L-Annamycin in acute myelogenous leukemia (AML) and metastatic soft tissue sarcoma (STS). Cardiac monitoring included serial electrocardiograms, cardiac biomarkers (troponin I/T), and transthoracic echocardiography with left ventricular ejection fraction (LVEF) and global longitudinal strain (GLS) assessment. All cardiac data were independently evaluated by a cardio-oncology laboratory at the Cleveland Clinic.

**Results:**

Ninety patients were included, with paired echocardiographic data available in 78. Median cumulative L-Annamycin dose was 660 mg/m^2^ (IQR 450–1,080). Baseline and post-treatment LVEF did not differ significantly (60.6% vs. 60%, *p* = 0.84). No associations were observed between change in LVEF and cumulative dose or age. Serial ECG, troponin, and GLS assessments showed no treatment-related cardiotoxicity. These findings were observed despite cumulative exposures exceeding conventional anthracycline lifetime limits.

**Conclusions:**

In this pooled analysis of patients with AML and STS, L-Annamycin was not associated with clinical or subclinical evidence of cardiotoxicity at cumulative doses exceeding traditional anthracycline thresholds. These data support continued clinical evaluation of L-Annamycin as a potentially safer anthracycline platform.

## Introduction

Anthracyclines are among the most effective and widely used chemotherapeutic agents. They have demonstrated consistent efficacy across a broad range of malignancies, including acute leukemias, lymphomas, soft tissue and bone sarcomas, Wilms tumor, neuroblastoma, and various metastatic solid tumors (1, 2). Despite their importance, their clinical use is limited by a cumulative, dose-dependent cardiotoxicity. Anthracycline-induced cardiac injury is mediated through multiple mechanisms, most notably topoisomerase IIβ inhibition and reactive oxygen species generation, resulting in cardiomyocyte death and progressive left ventricular dysfunction that may manifest years or even decades after exposure (3). This well-established risk has led to strict lifetime dose limitations, often restricting treatment intensity, limiting retreatment options, and excluding patients with prior anthracycline exposure or cardiovascular comorbidities from receiving potentially effective therapy (4).

**Figure 1:**
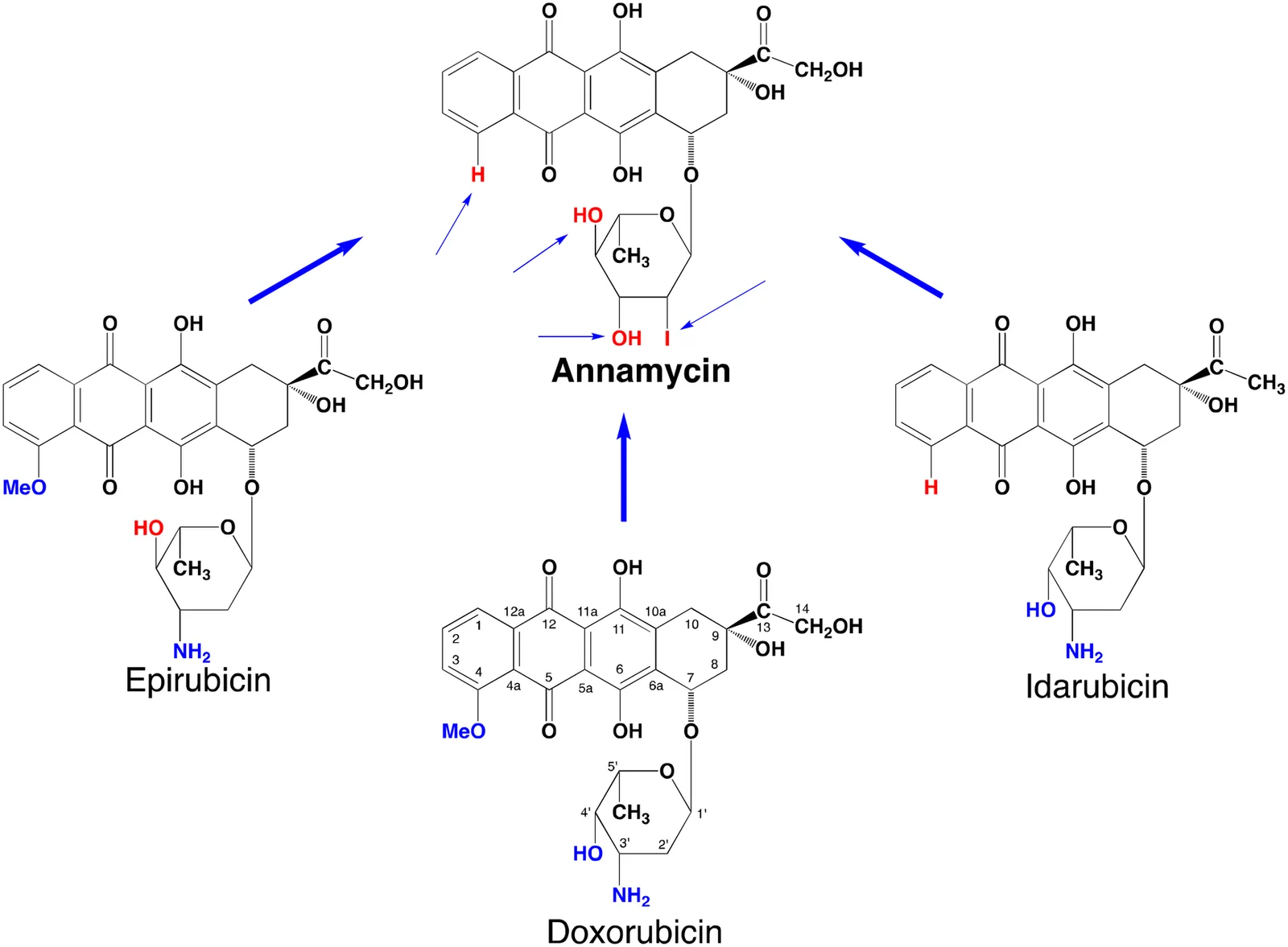
Structural modifications of annamycin relative to clinically used anthracyclines.

Annamycin is a next-generation anthracycline designed to maintain antitumor efficacy while addressing the key limitations of conventional agents. Structurally, it incorporates elements of doxorubicin, epirubicin, and idarubicin, with additional modifications including 3′-deamination and iodine substitution at the C-2 position (Figure 1). These changes preserve topoisomerase II inhibition but reduce cardiotoxic potential observed with traditional anthracyclines. Furthermore, development of a liposomal formulation (L-Annamycin) was intended to optimize tissue distribution and further minimize cardiac exposure (5).

Given the central role of anthracyclines in oncologic care, development of a less cardiotoxic agent is of substantial clinical importance. Preclinical studies have demonstrated markedly reduced cardiotoxicity of annamycin compared with doxorubicin while maintaining antitumor activity. We therefore sought to characterize the cardiac safety profile of L-Annamycin in patients treated at high cumulative doses.

## Materials and methods

### Study design and objectives

This study is a pooled analysis of cardiac safety data from five sponsor- and investigator-initiated clinical trials evaluating L-Annamycin. The trials included two studies in patients with metastatic soft tissue sarcoma (STS) and three studies in patients with acute myelogenous leukemia (AML). L-Annamycin was administered under multiple dosing and escalation regimens across indications.

The primary objective of this pooled analysis was to characterize the cardiac safety profile of L-Annamycin across varying dosing strategies and cumulative exposures.

### Clinical trials and treatment protocols

Five clinical trials evaluating L-Annamycin were included in this pooled analysis, with two studies in metastatic STS and three studies in AML. In four trials, L-Annamycin was administered as monotherapy. In one AML study, L-Annamycin was administered in combination with cytarabine (2,000 mg/m^2^/day for five consecutive days). Across all studies, L-Annamycin was administered in sequential dosing cohorts with dose escalation following completion of prior cohorts without dose-limiting toxicities.

In the sponsor-initiated STS study, L-Annamycin was administered once every three weeks until withdrawal of consent, unacceptable toxicity, or disease progression. Dosing began at 210 mg/m^2^ and escalated to 390 mg/m^2^. In the investigator-initiated STS study, L-Annamycin was administered weekly for three consecutive weeks (Days 1, 8, and 15) followed by one week off in a 28-day cycle. This study initiated with a dosing cohort of 35 mg/m^2^ and reached a final dosing cohort of 60 mg/m^2^.

The first AML trial evaluated L-Annamycin monotherapy at 100 mg/m^2^/day and 120 mg/m^2^/day administered for three consecutive days. The second AML monotherapy trial included five dosing cohorts ranging from 120 mg/m^2^/day to 240 mg/m^2^/day for three consecutive days, with repeat cycles permitted under predefined clinical criteria. The third AML study evaluated combination therapy with L-Annamycin (190 mg/m^2^/day or 230 mg/m^2^/day for three consecutive days) plus cytarabine for five consecutive days, with expansion of the 230 mg/m^2^ cohort following observed clinical activity. Repeat cycles were permitted in patients achieving remission when clinically appropriate.

A detailed summary of all the clinical trials included is shown in Table 1.

**Table 1:** Summary of L-Annamycin clinical trials included in the pooled safety analysis.

Study ID	Phase	Subjects included in independent cardiac safety review	Subjects included in paired LVEF analysis	Indication	L-Annamycin dose range (mg/m^2^/day)	Administration schedule	Combination therapy
MB-104	Phase 1	7	7	Relapsed/Refractory AML	100–120	IV Days 1–3 (single induction cycle)	Monotherapy
MB-105	Phase 1	20	19	Relapsed/Refractory AML	120–240	IV Days 1–3; 21-day cycles	Monotherapy
MB-106	Phase 1/2	22	18	AML (Relapsed/Refractory; previously untreated permitted in expansion phase)	190–230	IV Days 1–3; 21-day cycles	Cytarabine 2.0 g/m^2^/day (IV Days 1–5)
MB-107	Phase 1b/2	34	34	Soft Tissue Sarcoma (Pulmonary Metastases)	210–390	IV every 3 weeks (q3w)	Monotherapy
NIO-0002	Phase 1b/2 (IIT)	7	0	Soft Tissue Sarcoma (Pulmonary Metastases)	35–60	IV Days 1, 8, 15 (3 weeks on/1 week off)	Monotherapy

### Cardiac safety assessment

Cardiac safety monitoring was performed prospectively within each trial according to protocol-specified surveillance measures. Monitoring included serial 12-lead electrocardiograms (ECGs), measurement of cardiac biomarkers (troponin I and troponin T) using validated assays, and transthoracic echocardiography with assessment of left ventricular ejection fraction (LVEF) and global longitudinal strain (GLS). All evaluations were performed in a central laboratory at Duke University.

All available cardiac data, including ECGs, troponin measurements, and echocardiographic studies, were independently reviewed by a dedicated cardio-oncology laboratory at the Cleveland Clinic to determine whether treatment-related cardiotoxicity occurred. Data from all five trials were combined for the purpose of pooled cardiac safety analysis.

### Statistical analysis

**Figure 2:**
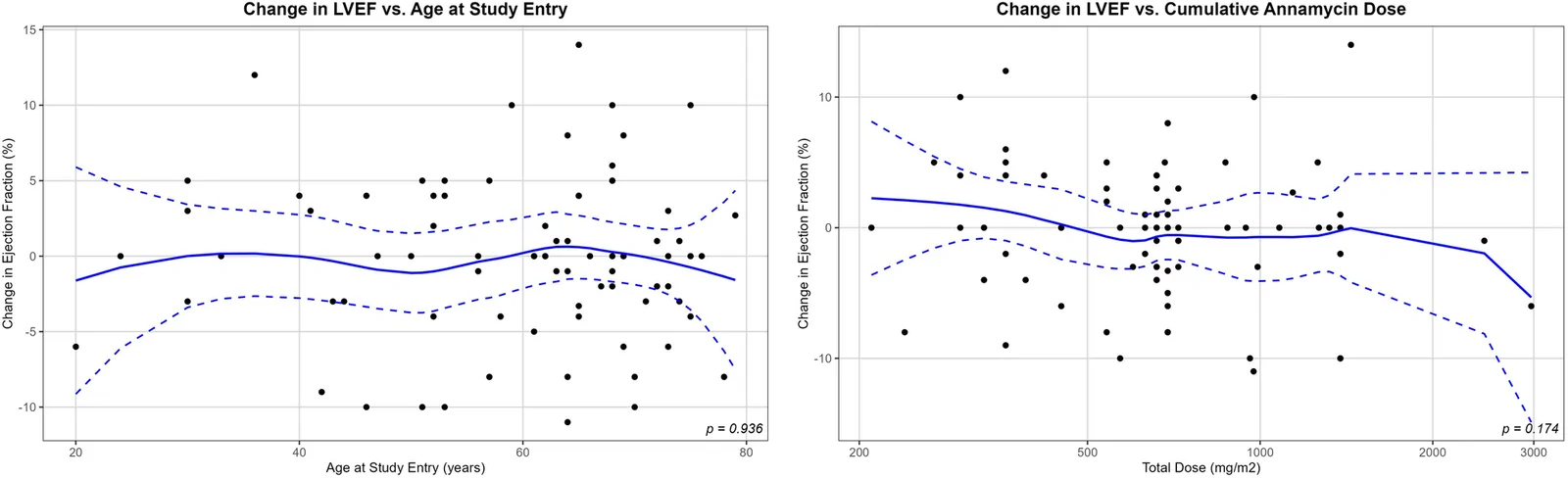
Scatterplots demonstrating the relationship between patient age (left) and cumulative L-Annamycin dose (right) with change in left ventricular ejection fraction from baseline to final assessment. The red solid line represents the linear regression fit with 95% confidence intervals (dashed lines).

Continuous variables are presented as mean ± standard deviation (SD), or median and interquartile range (IQR) as appropriate. Categorical variables are reported as frequencies and percentages. Continuous variables were assessed for normality using the Shapiro–Wilk test in conjunction with visual inspection of histogram distributions. A paired *t*-test was used to compare baseline and final ejection fraction measurements within the same patients. The association between change in LVEF and total cumulative dose of L-Annamycin was evaluated using linear regression analysis. A similar analysis was performed to assess the relationship between change in LVEF and patient age. Spearman rank correlation was utilized for these comparisons due to departures from normality among several variables and because relationships were not assumed to be strictly linear. Figure 2 was generated using R in RStudio with the ggplot2 package.

## Results

### Baseline characteristics

A total of 90 patients treated with L-Annamycin across the five trials were included and underwent independent cardio-oncology review. Five patients developed an allergic reaction to either L-Annamycin or cytarabine and were unable to continue therapy, and two were withdrawn due to deterioration in their overall clinical status. Source data-verified pre- and post-treatment ejection fraction measurements, along with other cardiac safety data, were available for 78 patients.

The median age at study entry was 64 years (IQR 53–70), and 41 patients (53%) were female. Prior anthracycline exposure was documented in 62 patients (79.5%), with the median prior exposure, expressed as daunorubicin-equivalent dose, being 330 mg/m^2^ (IQR 186–660; range 44–1,011.2 mg/m^2^). The median cumulative L-Annamycin dose received was 660 mg/m^2^ (IQR 450–1,080). Mean baseline left ventricular ejection fraction (LVEF) was 60.6 ± 5%, and the median interval between pre- and post-treatment echocardiograms was 53 days (IQR 30–107). Baseline cardiovascular risk factors included hypertension in 39 patients (50%), arrhythmias/condusction disorders in 24 (31%), valvular/structural disease in 18 (23%), thromboembolism in 18 (23%), dyslipidemia in 13 (17%), coronary artery disease in 7 (9%), aortic disease in 7 (9%), and diabetes mellitus in 7 (9%). A summary of the included patients’ baseline characteristics is shown in Table 2.

**Table 2:** Baseline characteristics of the paired cardiac safety population (*N* = 78).

Parameter	Value
Age, years	64 (53–70)
Female sex, *n* (%)	41 (52.6)
Race, *n* (%)	
White	71 (91.0)
Black or African American	2 (2.6)
Asian	2 (2.6)
Not reported	3 (3.8)
Ethnicity, *n* (%)	
Not Hispanic or Latino	46 (59.0)
Hispanic or Latino	5 (6.4)
Not collected/available	27 (34.6)
Prior anthracycline exposure, *n* (%)	62 (79.5)
Prior daunorubicin-equivalent dose, mg/m^2^	330 (186–660)
Baseline LVEF, %	60.6 ± 5.0
Cumulative L-Annamycin dose, mg/m^2^	660 (450–1,080)
Cardiovascular risk factors, *n* (%)	
Hypertension	39 (50.0)
Rhythm/conduction abnormalities	24 (30.8)
Valvular/structural heart disease	18 (23.1)
Thromboembolic disease/history	18 (23.1)
Lipid disorders	13 (16.7)
Coronary artery disease	7 (9.0)
Vascular/aortic disease	7 (9.0)
Diabetes mellitus	7 (9.0)
Baseline cardiovascular medications, *n* (%)	
Beta-blockers	21 (26.9)
ACE inhibitor/ARB	20 (25.6)
Antiplatelet/anticoagulants	15 (19.2)
Calcium channel blocker/nitrates	11 (14.1)
Statin/lipid-lowering therapy	10 (12.8)
Diuretics	6 (7.7)

Values are presented as median (interquartile range), mean ± standard deviation, or *n* (%), as appropriate.

### Cardiac safety evaluation

Baseline and post-treatment LVEF did not differ significantly (60.6% vs. 60%, *p* = 0.84). Moreover, no significant associations were observed between the change in LVEF and patient age or cumulative L-Annamycin dose, even when the cumulative dose reached over 1500 mg/m2: over 3 times the recommended maximum dose for anthracyclines. Correlation analyses demonstrated weak, non-significant relationships for both age (Spearman *ρ* −0.03, *p* = 0.73) and cumulative dose (Spearman *ρ* −0.17, *p* = 0.12) (Figure 2). Serial echocardiographic assessments, including centralized GLS analysis, did not demonstrate evidence of treatment-related myocardial dysfunction in these patients.

Regarding ECG abnormalities, there were 7 instances of tachycardia, none of which were life-threatening and all of which occurred in the setting of underlying clinical conditions or concurrent events. There were 3 transient cases of atrial fibrillation and one of atrial flutter. Three patients experienced transient QTc prolongation. None of these ECG changes were deemed to be serious by either the treating physicians or the consulting cardiologists and all but 2 (a transient tachycardia and a transient QTc prolongation) were classified as being unrelated to study drug.

Likewise, no consistent or clinically meaningful elevations in troponin I or troponin T were observed. Across the completed studies, 23 subjects had at least one post-treatment troponin-I or troponin-T value above the applicable central laboratory reference range where the corresponding baseline value for that analyte was not abnormal. None of the abnormal post-treatment troponin findings were assessed by the treating Investigator(s) as serious or related to study drug. One subject developed a significant troponin elevation several weeks after completion of L-Annamycin and cytarabine therapy. All available source data were independently reviewed, and based on temporal trends, serial troponin values, and the absence of supportive clinical or imaging findings suggestive of myocardial injury, this event was adjudicated as unrelated to study treatment, with a concomitant urinary tract infection deemed the more plausible explanation.

## Discussion

In this pooled analysis of five clinical trials, L-Annamycin was not associated with clinical or subclinical evidence of cardiotoxicity. Left ventricular ejection fraction remained stable, with no relationship between change in LVEF and cumulative dose or patient age, and no treatment-attributed abnormalities on ECG, troponin, or GLS assessment. Notably, these findings were observed despite cumulative doses that exceeded conventional lifetime anthracycline exposure limits.

Anthracyclines have long been associated with a dose-dependent risk of cardiotoxicity, with reported incidences of approximately 5% at 400 mg/m^2^ of doxorubicin, 26% at 550 mg/m^2^, and 48% at 700 mg/m^2^. However, no definitive safe dose threshold exists, as cardiotoxicity may occur even at cumulative doses below 100 mg/m^2^, particularly in patients with underlying cardiovascular risk factors (6, 7). More recently, liposomal anthracyclines have demonstrated improved cardiac safety. Pegylated liposomal doxorubicin (Doxil/Caelyx) reduced the risk of cardiac events more than threefold compared with conventional doxorubicin (HR 3.16, *P* < 0.001) (8), while non-pegylated liposomal doxorubicin (Myocet) reduced cardiotoxicity from 21% to 6% in one randomized trial (9), although available data suggest its cardioprotective effect may be less pronounced than that reported with Doxil (10). These limitations restrict treatment intensity and may preclude retreatment in high-risk populations despite the proven efficacy of anthracyclines. Preclinical investigations have demonstrated that Annamycin is associated with substantially reduced cardiotoxicity compared with doxorubicin. In chronic murine models, Annamycin exhibited markedly lower cardiac injury, attributed in part to differential tissue distribution and intracellular localization patterns (11, 12). Liposomal formulation further reduced cardiac exposure while increasing tumor drug accumulation, thereby improving tumor selectivity and therapeutic index (13). Moreover, L-Annamycin demonstrated potent anti-leukemic activity in treatment-naïve and drug-resistant AML models, showing synergy with cytarabine and venetoclax and durable disease control *in vivo*. In contrast to doxorubicin, it exhibited minimal cardiotoxicity in murine models and human cardiomyocytes, with preserved contractility and no histopathologic cardiac injury (14).

Beyond cardiac safety, L-Annamycin has shown a favorable efficacy profile. In AML, results from the MB-106 study demonstrated meaningful clinical activity in combination with cytarabine, with complete responses observed across lines of therapy, including in second-line and more advanced disease. Durable responses exceeding 12 months were noted in several patients, and some responders were successfully bridged to stem cell transplantation, supporting the potential role of L-Annamycin–based therapy in facilitating curative-intent treatment (15). These findings have supported continued clinical development, including an ongoing adaptive phase 3 trial in relapsed/refractory AML, which incorporates prospective acute and long-term cardiac safety monitoring with serial ECGs, echocardiography (including GLS where evaluable), and cardiac biomarkers as part of its predefined safety assessments.

Our study is the first to extend these preclinical observations into the clinical setting through an independent, centralized cardio-oncology adjudication across five trials and multiple dosing regimens. Importantly, no signal of clinical or subclinical cardiotoxicity was identified so far despite cumulative exposures exceeding conventional lifetime anthracycline limits. These findings suggest that L-Annamycin may allow administration of therapeutically meaningful doses without the cardiovascular limitation seen with traditional anthracyclines. If confirmed in larger, longer-term studies, this safety profile could expand treatment options for patients with prior anthracycline exposure, advanced age, or cardiovascular comorbidities who might otherwise be excluded from anthracycline-based therapy.

This study has several limitations. First, our analysis represents a pooled evaluation of early-phase trials without a randomized comparator arm, limiting direct comparison with conventional anthracyclines. Second, although cumulative doses exceeded traditional lifetime thresholds, the overall sample size remains modest, and follow-up duration was limited, precluding definitive conclusions regarding late-onset cardiotoxicity. Third, while comprehensive cardiac surveillance including LVEF, GLS, ECG, and biomarker assessment was performed, longer-term longitudinal monitoring will be necessary to fully characterize delayed cardiac effects. Therefore, these findings should be interpreted as hypothesis-generating and warrant confirmation in larger samples with extended follow-up.

## Conclusion

In this pooled analysis of five clinical trials in AML and metastatic soft tissue sarcoma, L-Annamycin was not associated with clinical or subclinical evidence of cardiotoxicity despite cumulative exposures exceeding conventional anthracycline limits. These findings support the continued clinical evaluation of L-Annamycin as a potentially safer anthracycline in malignancies where traditional agents are constrained by cardiac risk.

## Data Availability

Requests to access data analyzed in this study should be directed to the corresponding author colliep@ccf.org.
