# Supplementary material for: Cardiac safety of L-Annamycin: a pooled analysis of five clinical trials
Source: Front Cardiovasc Med. 2026 Sep 3;13:1817847. doi: 10.3389/fcvm.2026.1817847 (PMC13581671; doi:10.3389/fcvm.2026.1817847)
Supplement: Supplementary file 1 [file Datasheet1.pdf]

Table S1: Summary of All Investigator Reported TEAEs (i.e., Regardless of Assigned Relationship to Study Drug) for MB-104, MB-105, MB-106 and MB-107 – Cardiac Disorders and Investigations Relevant to Cardiac Dysfunction by Cumulative Dose at Time of AE Onset

System Organ Class<br>Preferred Term [1]	L-Annamycin Cumulative Dose at Time of AE Onset (mg/m²) [Subject ID No.]												
	**Partial Infusion**	**120**	**210**	**240**	**330**	**360**	**390**	**540**	**570**	**630**	**690**	**720**	**1,380**
**Cardiac disorders**	**3**	**0**	**1**	**1**	**0**	**2**	**1**	**0**	**0**	**1**	**2**	**2**	**1**
Acute myocardial infarction	1<br>[001-011]	-	-	-	-	-	-	-	-	-	-	-	-
Arrhythmia	-	-	-	-	-	-	-	-	-	1<br>[48005-012]	-	-	-
Atrial fibrillation	-	-	-	-	-	-	-	-	-	-	2<br>[102-002]<br>[106-003]	1<br>[48005-019]	-
Atrial flutter	-	-	-	-	-	-	-	-	-	-	-	-	1<br>[203-003]
Left ventricular dysfunction	1<br>[001-011][a]	-	-	-	-	-	-	-	-	-	-	-	-
Sinus tachycardia	-	-	-	1<br>[48005-013]	-	-	1<br>[001-004]	-	-	-	-	-	-
Tachycardia	1<br>[48001-007][b]	-	1<br>[48005-010]	-	-	2<br>[004-005]<br>[48004-002]	-	-	-	-	-	1<br>[48005-017]	-
**Investigations (relevant to cardiac dysfunction)**	**0**	**1**	**0**	**0**	**1**	**0**	**0**	**1**	**1**	**0**	**1**	**0**	**0**
ECG signs of myocardial ischaemia	-	-	-	-	1<br>[001-006]	-	-	-	-	-	-	-	-
Electrocardiogram QT prolonged	-	1<br>[48004-002]	-	-	-	-	-	1<br>[48002-009]	1<br>[201-001]	-	-	-	-
Electrocardiogram T Wave alternans	-	-	-	-	-	-	-	-	-	-	1<br>[201-003]	-	-

a Subject assigned to 330 mg/m2/day dose level experienced a severe (Grade 3) infusion related reaction upon administration of the first dose of L-Annamycin. The study drug infusion was stopped approximately 1 minute after the start of infusion and the subject was not rechallenged with L-Annamycin. Two days later, the subject experienced life-threatening (Grade 4) acute respiratory failure and was intubated. During the time that the subject was hospitalized for these serious adverse events (SAEs), moderate (Grade 2) acute myocardial infarction and severe (Grade 3) left ventricular dysfunction were observed

b Subject assigned to 180 mg/m2/day dose level experienced a moderate (Grade 2) anaphylactic reaction on Day 1 after a partial infusion of L-Annamycin. The study drug infusion was temporarily stopped. Mild tachycardia occurred after the subject received the partial infusion of study drug.

To supplement the dose-at-onset summary above and directly address the reviewer's request for severity, patient-level details for each subject/preferred-term entry are provided in Table 3 below. This table preserves the same scope as the summary table (investigator-reported treatment-emergent Cardiac disorders and Investigations terms relevant to cardiac dysfunction, regardless of relationship to study drug) and adds severity, seriousness, outcome, and key clinical context.

Table S2: Patient-Level Details for Treatment-Emergent Cardiac and ECG/Investigation Findings Relevant to Cardiac Dysfunction

Study	Subject ID	Category	Preferred term	Event(s) / severity	PI-Reported Relatedness to Study Drug	Serious?	Outcome / key clinical context
MB-104	004-005	Cardiac disorders	Tachycardia	1; Grade 3	Unrelated	No	Recovered without sequelae in 14 days with standard therapy; no study-drug interruption/discontinuation due to event;
MB-105	48004-002	Cardiac disorders	Tachycardia	2; Grade 1 and Grade 2	Unrelated/Unrelated	No	Both recovered same day without treatment; no study-drug interruption/discontinuation;
MB-105	48001-007	Cardiac disorders	Tachycardia	2; Grade 2 and Grade 1	Probably/Possibly	No	Occurred Day 1 with Grade 2 anaphylactic reaction; both resolved; subject completed Day 1 dosing, Cycle 1 Days 2/3, and a second cycle without further tachycardia;.
MB-105	48005-010	Cardiac disorders	Tachycardia	1; Grade 1	Unrelated	No	Occurred Day 2 before infusion after prior Day 1 dose; recovered without sequelae in 7 days with standard therapy; no interruption/discontinuation;
MB-105	48005-017	Cardiac disorders	Tachycardia	1; Grade 2	Unlikely	No	Occurred 1 day after last dose; recovered without sequelae in 3 days without treatment; no interruption/discontinuation;
MB-105	48005-012	Cardiac disorders	Arrhythmia	1; Grade 2	Unrelated	No	Occurred 11 days after last dose; event recovering/resolving at study termination; no interruption/discontinuation;
MB-105	48005-019	Cardiac disorders	Atrial fibrillation	1; Grade 4	Unrelated	No	Occurred with severe infectious/allergic complications, Grade 4 laryngeal oedema, and Multi organ dysfunction syndrome related to progressive AML;
MB-105	48005-013	Cardiac disorders	Sinus tachycardia	1; Grade 1	Unrelated	No	Occurred Day 1 after one infusion; recovered without sequelae in 2 days without treatment; no interruption/discontinuation; Grade 1 sinus tachycardia reported at baseline.
MB-105	48004-002	Investigations	Electrocardiogram QT prolonged	2; Grade 2 and Grade 1	Definitely/Probably	No	Grade 2 after 120 mg/m2; Grade 1 after 360 mg/m2; both recovered same day without treatment. Site used Bazett rather than Fridericia formula to calculate QTc
MB-105	48002-009	Investigations	Electrocardiogram QT prolonged	1; Grade 1	Unrelated	No	Occurred 19 days after last dose; recovered same day without treatment; no interruption/discontinuation;
MB-106	102-002	Cardiac disorders	Atrial fibrillation	1; Grade 2	Unrelated	No	Occurred 18 days after last dose; recovered same day with standard therapy; no interruption/discontinuation;
MB-106	106-003	Cardiac disorders	Atrial fibrillation	1; Grade 3	Unrelated	No	Occurred 18 days after last dose; ongoing at study completion/death due to AML progression; no interruption/discontinuation;
MB-106	203-003	Cardiac disorders	Atrial flutter	1; Grade 2	Unrelated	No	Occurred 3 days after last dose; recovered without sequelae in 2 days with standard therapy; no interruption/discontinuation;
MB-106	201-001	Investigations	Electrocardiogram QT prolonged	1; Grade 2	Unrelated	No	Occurred 18 days after Cycle 1 last dose; recovered without sequelae in 7 days without treatment; no interruption/discontinuation;
MB-106	201-003	Investigations	Electrocardiogram T-wave alternans	1; Grade 1	Unrelated	No	Occurred 11 days after last dose; recovered without sequelae in 1 day without treatment; no interruption/discontinuation;

Study	Subject ID	Category	Preferred term	Event(s) / severity	PI-Reported Relatedness to Study Drug	Serious?	Outcome / key clinical context
MB-107	001-011	Cardiac disorders	Acute myocardial infarction	1; Grade 2	Unrelated	No	Observed during hospitalization after Grade 3 infusion-related reaction and Grade 4 acute respiratory failure; study drug stopped due to infusion reaction; PI attributed event to be associated with acute respiratory failure possibly caused by fluid overload;
MB-107	001-011	Cardiac disorders	Left ventricular dysfunction	1; Grade 3	Unrelated	No	Observed during same hospitalization after infusion-related reaction and acute respiratory failure; study drug stopped due to infusion reaction; PI attributed event to be associated with acute respiratory failure possibly caused by fluid overload
MB-107	001-004	Cardiac disorders	Sinus tachycardia	1; Grade 1	Unrelated	No	Occurred 8 days after last dose; ongoing at study completion; no interruption/discontinuation; PI attributed to concurrent anemia;
MB-107	001-006	Investigations	ECG signs of myocardial ischaemia	1; Grade 2	Unrelated	No	Occurred 24 days after Cycle 1 infusion; recovered same day with standard therapy; no interruption/discontinuation; Cycle 2 given same day;

Events assessed by Investigators as possibly, probably, or definitely related to L-Annamycin were independently reviewed in the context of available cardiac safety data. For Subject 48004-002, the reported QT prolongation events were reviewed after it was confirmed that the site used the Bazett rather than Fridericia correction formula, and the findings were not considered evidence of L-Annamycin-induced cardiotoxicity. For Subject 48001-007, the tachycardia events occurred in the setting of a concurrent Grade 2 anaphylactic reaction, and independent review likewise did not support evidence of L-Annamycin-induced cardiotoxicity. Across the events summarized, review of the available echocardiographic and cardiac biomarker data did not identify evidence of L-Annamycin-induced cardiotoxicity.

To provide the requested troponin context, subject-level listings of all available central laboratory troponin-I and troponin-T results are provided below for MB-104, MB-105, MB-106, and MB-107. These tables include available troponin results by subject, collection timepoint, and cumulative L-Annamycin dose at the time of collection. The troponin-evaluable population may differ from the paired LVEF population, as troponin availability depended on protocol-specified collection requirements, sample availability, and assay/reporting availability within each study.

The listings below provide the full value ranges by study, analyte, subject, collection timepoint, and cumulative L-Annamycin dose, with abnormal values flagged relative to the applicable central laboratory reference range. The number of available measurements and number of subjects with values above the applicable reference range vary by study because troponin collection schedules, assay methodology, and sample availability differed across the completed studies. Across the completed studies, 23 subjects had at least one post-treatment troponin-I or troponin-T value above the applicable central laboratory reference range where the corresponding baseline value for that analyte was not abnormal. None of the abnormal post-treatment troponin findings were assessed by the treating Investigator(s) as related to study drug.

Table S3: Subject Listing of Troponin-T and Troponin I Results for Study MB-104

SUBJECT NO.	DOSE LEVEL (MG/M^2/DAY) [a]	TROPONIN-T (µg/L) [b]			TROPONIN-I (ng/mL) [b]		
		D1 Pre-dose	Day 3 (CAD)	Day 21 (CAD)	D1 Pre-dose	Day 3 (CAD)	Day 21 (CAD)
**005-004**	100	<0.01	<0.01 (300 mg/m^2)	<0.01 (300 mg/m^2)	<0.30	<0.30 (300 mg/m^2)	<0.30 (300 mg/m^2)
**006-001**	100	ND	ND	ND	ND	ND	ND
**006-002**	100	ND	ND	ND	ND	ND	ND
**006-003**	100	<0.01	<0.01 (300 mg/m^2)	<0.01 (300 mg/m^2)	<0.30	<0.30 (300 mg/m^2)	<0.30 (300 mg/m^2)
**004-005**	120	<0.01	<0.01 (360 mg/m^2)	ND	<0.30	<0.30 (360 mg/m^2)	ND
**004-006**	120	<0.01	<0.01 (360 mg/m^2)	<0.01 (360 mg/m^2)	<0.30	<0.30 (360 mg/m^2)	<0.30 (360 mg/m^2)
**006-007**	120	0.01	<0.01 (360 mg/m^2)	<0.01 (360 mg/m^2)	<0.30	<0.30 (360 mg/m^2)	<0.30 (360 mg/m^2)

CAD = cumulative L-Annamycin for Injection dose at the time of lab draw; ND = not done [blood sample collection for troponin-T and troponin-I testing were not done either due to site error or because troponin testing had not been incorporated into the protocol version approved at the time of testing]

[a] Administered daily for three consecutive days

[b] Troponin testing performed at ICON Central Lab (Normal reference ranges: troponin T <0.01 µg/L; troponin I <0.31 ng/mL). Per protocol, Day 1 troponin-T and troponin-I collections occurred pre-dose on Day 1, at the end of the 2-hour L-Annamycin for Injection

infusion on Day 3, and at any point during the Day 21 visit (MB-104 only required L-Annamycin monotherapy dosing over 3 consecutive days [Day 1 through Day 3]). As such, all Day 21 troponin-T and -I values, respectively, reflect troponin levels ~18 days following the last dose of L-Annamycin for Injection on Day 3.

Table S4: Subject Listing of Troponin-T and Troponin I Results for Study MB-105

Subject ID	Dose Level ($mg/m^2/day$)[a]	Troponin-T (µg/L)[b]			Troponin-I (ng/mL)[b]		
		Day 1 Pre-dose (CAD)	Day 3 (CAD)	Day 21 (CAD)	Day 1 Pre-dose (CAD)	Day 3 (CAD)	Day 21 (CAD)
48001-001	120	<0.01	<0.01 (360 mg/m^2)	ND	<0.30	<0.30 (360 mg/m^2)	ND
48004-002	120	<0.01	<0.01 (360 mg/m^2)	ND	<0.30	<0.30 (360 mg/m^2)	ND
48001-003	120	<0.01	<0.01 (360 mg/m^2)	ND	<0.30	<0.30 (360 mg/m^2)	ND
48004-004	150	<0.01	<0.01 (450 mg/m^2)	<0.01 (450 mg/m^2)	<0.30	<0.30 (450 mg/m^2)	<0.30 (450 mg/m^2)
48004-005	150	<0.01	<0.01 (450 mg/m^2)	<0.01 (450 mg/m^2)	<0.30	<0.30 (450 mg/m^2)	<0.30 (450 mg/m^2)
48004-006	150	<0.01	<0.01 (450 mg/m^2)	<0.01 (450 mg/m^2)	<0.30	<0.30 (450 mg/m^2)	<0.30 (450 mg/m^2)
48001-007	180	<0.01	<0.01 (540 mg/m^2)	<0.01 (540 mg/m^2)	<0.30	<0.30 (540 mg/m^2)	<0.30 (540 mg/m^2)
48001-008	180	<0.01	<0.01 (540 mg/m^2)	ND	<0.30	<0.30 (540 mg/m^2)	ND
48002-009	180	<0.01	<0.01 (540 mg/m^2)	<0.01 (540 mg/m^2)	<0.30	<0.30 (540 mg/m^2)	<0.30 (540 mg/m^2)
48005-010	210	<0.01	<0.01 (630 mg/m^2)	<0.01 (630 mg/m^2)	<0.30	<0.30 (630 mg/m^2)	<0.30 (630 mg/m^2)
48005-011	210	<0.01	<0.01 (630 mg/m^2)	<0.01 (630 mg/m^2)	<0.30	<0.30 (630 mg/m^2)	<0.30 (630 mg/m^2)
48005-012	210	<0.01	<0.01 (630 mg/m^2)	<0.01 (630 mg/m^2)	<0.30	<0.30 (630 mg/m^2)	<0.30 (630 mg/m^2)
48005-013	240	0.01	<0.01 (720 mg/m^2)	<0.01 (720 mg/m^2)	<0.30	<0.30 (720 mg/m^2)	<0.30 (720 mg/m^2)
48006-014[c]	240	ND	ND	ND	ND	ND	ND

Subject ID	Dose Level (mg/m²/day)[a]	Troponin-T (µg/L)[b]			Troponin-I (ng/mL)[b]		
		Day 1 Pre-dose (CAD)	Day 3 (CAD)	Day 21 (CAD)	Day 1 Pre-dose (CAD)	Day 3 (CAD)	Day 21 (CAD)
48006-015	240	<0.01	<0.01 (720 mg/m^2)	<0.01 (720 mg/m^2)	<0.30	<0.30 (720 mg/m^2)	<0.30 (720 mg/m^2)
48006-016[c]	240	<0.01	ND	<0.01 (240 mg/m^2)	<0.30	ND	<0.30 (240 mg/m^2)
48005-017	240	<0.01	<0.01 (720 mg/m^2)	<0.01 (720 mg/m^2)	<0.30	<0.30 (720 mg/m^2)	<0.30 (720 mg/m^2)
48006-018 (Cycle 1)	240	<0.01	<0.01 (720 mg/m^2)	<0.01 (720 mg/m^2)	<0.30	<0.30 (720 mg/m^2)	<0.30 (720 mg/m^2)
48006-018 (Cycle 2)	240	<0.01 (720 mg/m^2)	<0.01 (1440 mg/m^2)	<0.01 (1440 mg/m^2)	<0.30 (720 mg/m^2)	<0.30 (1440 mg/m^2)	<0.30 (1440 mg/m^2)
48005-019	240	<0.01	<0.01 (720 mg/m^2)	<0.01 (720 mg/m^2)	<0.30	<0.30 (720 mg/m^2)	<0.30 (720 mg/m^2)
48005-020[c]	240	<0.01	ND	ND	<0.30	ND	ND

CAD= cumulative L-Annamycin for Injection dose at the time of lab draw ND = not done [Troponin T/I testing was not done either due to site error or unexpected courier delays (resulting in samples being received unfrozen]

[a] Administered daily for three consecutive days

[b] Troponin testing performed at ICON Central Lab (Normal reference ranges: troponin T <0.01 µg/L; troponin I <0.31 ng/mL). Per protocol, Day 1 troponin-T and troponin-I collections occurred pre-dose on Day 1, at the end of the 2-hour L-Annamycin for Injection infusion on Day 3, and at any point during the Day 21 visit (MB-105 only required L-Annamycin monotherapy dosing over 3 consecutive days [Day 1 through Day 3]). As such, all Day 21 troponin-T and -I values, respectively, reflect troponin levels ~18 days following the last dose of L-Annamycin for Injection on Day 3.

[c] Subject did not receive full 3 days of L-Annamycin due to non-cardiac related toxicities.

Table S5: Subject Listing of Troponin-T and Troponin-I Results for Study MB-106

SUBJECT NO./ GENDER	DOSE LEVEL MG/M²/ DAY [a]	TROPONIN-T (ng/mL)[b]						TROPONIN-I (ng/mL)[b]					
		D1 PRE-DOSE	DAY 3 (CAD)	DAY 21 (CAD)	C2D1 (CAD)	C2D3 (CAD)	C2D21 (CAD)	D1 PRE-DOSE	DAY 3 (CAD)	DAY 21 (CAD)	C2D1 (CAD)	C2D3 (CAD)	C2D21 (CAD)
**201-001/M**	190	13	17 (570 mg/m²)	29* (570 mg/m²)	13[c] (1140 mg/m²)	16[c] (1710 mg/m²)	18[c] (1710 mg/m²)	4.6	7.8 (570 mg/m²)	7.8 (570 mg/m²)	<3.5[c] (1140 mg/m²)	6.3[c] (1710 mg/m²)	4.6[c] (1710 mg/m²)
**202-001/M**	190	8.0	<6.0 (570 mg/m²)	7.0 (570 mg/m²)	N/A	N/A	N/A	4.7	4.1 (570 mg/m²)	<3.5 (570 mg/m²)	N/A	N/A	N/A
**103-001/M**	190	ND	ND	22 (570 mg/m²)	N/A	N/A	N/A	ND	ND	21.1 (570 mg/m²)	N/A	N/A	N/A
**201-002/F**	230	8.0	37* (690 mg/m²)	ND	N/A	N/A	N/A	8.4	50* (690 mg/m²)	ND	N/A	N/A	N/A
**102-001/M**	230	65*	ND	ND	N/A	N/A	N/A	3.6	ND	ND	N/A	N/A	N/A
**201-003/M**	230	46*	30* (690 mg/m²)	42* (690 mg/m²)	N/A	N/A	N/A	6.1	6.6 (690 mg/m²)	11.1 (690 mg/m²)	N/A	N/A	N/A
**104-001/M**	230	14	13 (690 mg/m²)	19 (690 mg/m²)	N/A	N/A	N/A	5.2	7.3 (690 mg/m²)	4.2 (690 mg/m²)	N/A	N/A	N/A
**102-002/F**	230	8	12 (690 mg/m²)	ND	N/A	N/A	N/A	4.8	11.0 (690 mg/m²)	ND	N/A	N/A	N/A
**104-002/M**	230	7	13 (690 mg/m²)	7 (690 mg/m²)	<6.0 (690 mg/m²)	<6.0 (1380 mg/m²)	11.0 (1380 mg/m²)	<3.5	24.3 (690 mg/m²)	ND (690 mg/m²)	<3.5 (690 mg/m²)	<3.5 (1380 mg/m²)	<3.5 (1380 mg/m²)
**104-003/F**	230	7	8 (690 mg/m²)	9 (690 mg/m²)	6 (690 mg/m²)	8 (1380 mg/m²)	8 (1380 mg/m²)	<3.5	3.5 (690 mg/m²)	4.8 (690 mg/m²)	4.5 (690 mg/m²)	<3.5 (1380 mg/m²)	4.1 (1380 mg/m²)

SUBJECT NO./ GENDER	DOSE LEVEL MG/M²/ DAY [a]	TROPONIN-T (ng/mL)[b]						TROPONIN-I (ng/mL)[b]					
		D1 PRE-DOSE	DAY 3 (CAD)	DAY 21 (CAD)	C2D1 (CAD)	C2D3 (CAD)	C2D21 (CAD)	D1 PRE-DOSE	DAY 3 (CAD)	DAY 21 (CAD)	C2D1 (CAD)	C2D3 (CAD)	C2D21 (CAD)
**201-004/F**	230	10	ND	ND	ND	ND	ND	4.1	ND	ND	ND	ND	ND
**104-004/F**	230	6	8 (690 mg/m²)	8 (690 mg/m²)	N/A	N/A	N/A	4.8	3.6 (690 mg/m²)	12.7 (690 mg/m²)	N/A	N/A	N/A
**203-001/F**	230	9	10 (690 mg/m²)	15* (690 mg/m²)	N/A	N/A	N/A	<3.5	6.5 (690 mg/m²)	12.4 (690 mg/m²)	N/A	N/A	N/A
**104-005/F**	230	7	11 (690 mg/m²)	122* (690 mg/m²)	N/A	N/A	N/A	<3.5	6.2 (690 mg/m²)	323* (690 mg/m²)	N/A	N/A	N/A
**203-002/M**	230	11	8 (690 mg/m²)	7 (690 mg/m²)	14 (690 mg/m²)	10 (1380 mg/m²)	8 (1380 mg/m²)	<3.5	3.7 (690 mg/m²)	<3.5 (690 mg/m²)	7.5 (690 mg/m²)	8.1 (1380 mg/m²)	3.5 (1380 mg/m²)
**103-002/F**	230	18*	16* (690 mg/m²)	16* (690 mg/m²)	125* (690 mg/m²)	110* (1380 mg/m²)	67* (1380 mg/m²)	13.1	17 (690 mg/m²)	14.2 (690 mg/m²)	464.8* (690 mg/m²)	333.6* (1380 mg/m²)	173.8* (1380 mg/m²)
**203-003/M**	230	10	11 (690 mg/m²)	18 (690 mg/m²)	14 (690 mg/m²)	16 (1380 mg/m²)	26* (1380 mg/m²)	3.8	6.6 (690 mg/m²)	12.7 (690 mg/m²)	15.6 (690 mg/m²)	17.1 (1380 mg/m²)	18.9 (1380 mg/m²)
**103-003/F**	230	8	11 (690 mg/m²)	15* (690 mg/m²)	N/A	N/A	N/A	5.1	6.3 (690 mg/m²)	7.5 (690 mg/m²)	N/A	N/A	N/A
**106-001/M**	230	<6	<6 (690 mg/m²)	ND	N/A	N/A	N/A	<3.5	<3.5 (690 mg/m²)	ND	N/A	N/A	N/A
**106-002/F**	230	10	7 (690 mg/m²)	14 (690 mg/m²)	11 (690 mg/m²)	8 (1380 mg/m²)	10 (1380 mg/m²)	<3.5	<3.5 (690 mg/m²)	<3.5 (690 mg/m²)	<3.5 (690 mg/m²)	<3.5 (1380 mg/m²)	<3.5 (1380 mg/m²)
**106-003/M**	230	9	6 (690 mg/m²)	16 (690 mg/m²)	N/A	N/A	N/A	3.6	4.6 (690 mg/m²)	6 (690 mg/m²)	N/A	N/A	N/A
**106-004/M**	230	30*	21 (690 mg/m²)	31* (690 mg/m²)	N/A	N/A	N/A	13.8	13.2 (690 mg/m²)	12.7 (690 mg/m²)	N/A	N/A	N/A

CAD= cumulative L-Annamycin for Injection dose at the time of lab draw; F = female; M = male; N/A = not applicable as troponin assessments were not performed because subject did not receive additional cycle; ND = troponin-T or -I testing not done at ICON due to investigational site error

[a] L-Annamycin for Injection dose administered daily for 3 consecutive days in combination with Cytarabine Injection at 2.0 g/m²/day for 5 consecutive days

[b] Troponin testing performed at ICON Central Lab. Normal reference ranges (M): troponin-T: ≤ 22 ng/mL; troponin-I: ≤ 35 ng/mL. Normal reference Ranges (F): troponin-T: ≤ 14 ng/mL; troponin-I: ≤ 17 ng/mL. Per protocol, blood samples were collected at Day 1 (pre-dose), end of L-Annamycin for Injection infusion on Cycle 1 Day 3 (within 2 hours after completion of the infusion), and Cycle 1 Day 21. If an additional Cycle was administered, samples were also collected on Days 1, 3, and 21 of the additional cycle at the same timepoints.

[c] Troponin-T and Troponin-I values reflected in the table are from the subject's 3rd cycle of L-Annamycin for Injection (2nd cycle was administered at the same dose level [190 mg/m^2/day] but administered under compassionate use as the approved version of the protocol in Italy at the time did not permit additional cycles of study drug). The cumulative L-Annamycin for Injection doses reported in the table includes L-Annamycin exposure from the compassionate use cycle (Cycle 2) in addition to the L-Annamycin exposure from Cycle 3 (administered on study).

* Indicates troponin value outside of normal range. Those highlighted in yellow (i.e., for subject 103-002) were assessed as clinically significant and are discussed below. All others were deemed non-clinically significant by treating Investigator.

Subject 103-002, the one subject with reported abnormal troponin-I and -T values deemed clinically significant by the treating Investigator, presented on study (pre-study drug treatment) with slightly elevated troponin-T values (18 ng/mL), which remained slightly elevated at the post-treatment troponin assessment timepoints (16 ng/mL at Day 3 and 16 ng/mL at Day 21). These elevated troponin-T values were assessed as non-clinically significant. This subject was noted to have normal troponin-I values prior to treatment on study, as well as at both post-treatment troponin collection timepoints during her first cycle of therapy. At the Cycle 2 Day 1 pre-dose timepoint, approximately 47 days from the last dose of L-Annamycin for Injection received during Cycle 1, significant increases in both troponin-I and troponin-T were observed and assessed as clinically significant. At both post-Cycle 2 treatment timepoints (i.e., Cycle 2 Day 3 and Cycle 2 Day 21), the troponin-I and troponin-T values showed a downward trend but remained elevated and were assessed as clinically significant by the treating Investigator. All available data (e.g., prior therapy, medical history, concomitant medications, AE/SAE reports, on study treatment details, and LVEF results for ECHOs performed on study including GLS % reported by Duke ECHO Core Lab) for this subject were sent for an independent case review by an expert in anthracycline-induced cardiotoxicity at the Cleveland Clinic. Based on the temporal trends observed in this case, the serial values of troponins observed during Cycle 2, and the lack of additional clinical and imaging evidence suggestive of cardiac damage, it was concluded that this troponin-I/-T elevation was unrelated to the combination drug product therapy with a more plausible clinical explanation being a concomitant illness (urinary tract infection).

Table S6: Subject Listing of Troponin-T and Troponin-I Results for Study MB-107

Subject No/ Gender	Dose Level mg/m^2 /day[a]	TROPONIN-I/TROPONIN-T (ng/mL)[b]																					
		SCREENING		CYC1 (CAD MG/M^2)		CYC2 (CAD MG/M^2)		CYC3 (CAD MG/M^2)		CYC4 (CAD MG/M^2)		CYC5 (CAD MG/M^2)		CYC6 (CAD MG/M^2)		CYC7 (CAD MG/M^2)		CYC8 (CAD MG/M^2)		CYC9 (CAD MG/M^2)		EOT (CAD MG/M^2)	
		I	T	I	T	I	T	I	T	I	T	I	T	I	T	I	T	I	T	I	T	I	T
**002-001/ F**	210	<3.5	<6.0	<3.5	<6.0	5.9<br>(420)	6.0<br>(420)	<3.5<br>(630)	<6.0<br>(630)	4.4<br>(840)	9.0<br>(840)	ND	9.0<br>(1050)	5.2<br>(1260)	10.0<br>(1260)	-	-	-	-	-	-	4.2<br>(1260)	10.0<br>(1260)
**002-003/ M**	210	86<br>*	8.0	44.4<br>*	9.0	-	-	-	-	-	-	-	-	-	-	-	-	-	-	-	-	79.0<br>*<br>(210)	15.0<br><br>(210)
**002-004/ M**	210	4.1	11	4.1	8.0	4.8<br>(420)	9.0<br>(420)	6.6<br>(630)	13<br>(630)	4.1<br>(840)	7.0<br>(840)	6.4<br>(1050)	11.0<br>(1050)	4.2<br>(1320)	8.0<br>(1320)	-	-	-	-	-	-	5.1<br>(1320)	11.0<br>(1320)
**002-005/ M**	270	<3.5	<6.0	ND	<6.0	<3.5<br>(540)	<6.0<br>(540)	3.6<br>(742)	< 6.0<br>(742)	<3.5<br>(944)	< 6.0<br>(944)	-	-	-	-	-	-	-	-	-	-	<3.5<br>(944)	<6.0<br>(944)
**002-006/ F**	270	8.9	10	ND	10	10.5<br>(540)	8.0<br>(540)	-	-	-	-	-	-	-	-	-	-	-	-	-	-	39.9<br>*<br>(540)	33.0<br>*<br>(540)
**002-007/ F**	270	<3.5	<6.0	ND	<6.0	-	-	-	-	-	-	-	-	-	-	-	-	-	-	-	-	5.2<br>(270)	8.0<br>(270)
**002-008/ F**	330	< 3.5	< 6.0	4.0	< 6.0	-	-	-	-	-	-	-	-	-	-	-	-	-	-	-	-	6.1<br>(330)	10.0<br>(330)
**003-001/ F**	330	ND	8.0	ND	12.0	ND	18.0<br>*<br>(660)	-	-	-	-	-	-	-	-	-	-	-	-	-	-	ND<br>(660)	14.0<br>(660)
**003-002/ M**	330	ND	10.0	ND	12.0	ND	15.0<br>(660)	-	-	-	-	-	-	-	-	-	-	-	-	-	-	ND	16.0<br>(660)
**002-010/ M**	390	4.7	15.0	4.7	16.0	23.3<br>(683)	27.0<br>*<br>(683)	7.7<br>(976)	34.0<br>*<br>(976)	-	-	-	-	-	-	-	-	-	-	-	-	11.8<br>(976)	27.0<br>*<br>(976)
**002-012/ F**	390	<3.5	< 6.0	< 3.5	< 6.0	<3.5<br>(682)	<6.0<br>(682)	-	-	-	-	-	-	-	-	-	-	-	-	-	-	10.4<br>(682)	13.0<br>(682)
**002-013/ F**	390	10.7	16.0	7.7	14.0	3.7<br>(682)	11.0<br>(682)	20.9<br>*<br>(877)	23.0<br>*<br>(877)	-	-	-	-	-	-	-	-	-	-	-	-	ND	ND
**003-003/ M**	390	ND	ND	3.9	14.0	<3.5<br>(682)	9.0<br>(682)	ND<br>(972)	ND<br>(972)	4.4<br>(1266)	11.0<br>(1266)	-	-	-	-	-	-	-	-	-	-	ND	ND
**001-003/ F**	210	<3.5	34<br>*	ND	ND	3.5<br>(540)	10.0<br>(540)	-	-	-	-	--	-	-	-	-	-	-	-	-	-	9.6<br>(540)	16<br>*<br>(540)
**004-001/ F**	390	29.6<br>*	11	50.3<br>*	15<br>*	34<br>*<br>(682)	20<br>*<br>(682)	ND	ND	-	-	-	-	-	-	-	-	-	-	-	-	69.4<br>*<br>(974)	22<br>*<br>(974)
**001-004/ F**	390	<3.5	9.0	ND	ND	-	-	-	-	-	-	-	-	-	-	-	-	-	-	-	-	12.7<br>(390)	25.0<br>*<br>(390)
**003-004/ F**	360	< 3.5	7.0	< 3.5	8.0	5.4<br>(720)	19<br>*<br>(720)	-	-	-	-	-	-	-	-	-	-	-	-	-	-	4.0<br>(720)	16<br>*<br>(720)

Subject No/ Gender	Dose Level mg/m^2 /day[a]	TROPONIN-I/TROPONIN-T (ng/mL)[b]																					
		SCREENING		CYC1 (CAD MG/M^2)		CYC2 (CAD MG/M^2)		CYC3 (CAD MG/M^2)		CYC4 (CAD MG/M^2)		CYC5 (CAD MG/M^2)		CYC6 (CAD MG/M^2)		CYC7 (CAD MG/M^2)		CYC8 (CAD MG/M^2)		CYC9 (CAD MG/M^2)		EOT (CAD MG/M^2)	
		I	T	I	T	I	T	I	T	I	T	I	T	I	T	I	T	I	T	I	T	I	T
**005-001/ F**	360	7.4	<6.0	ND	ND	<3.5<br>(690)	11.0<br>(690)	-	-	-	-	-	-	-	-	-	-	-	-	-	-	22.2<br>*<br>(690)	32<br>*<br>(690)
**005-002/ M**	360	5.4	73<br>*	ND	118<br>*	-	-	-	-	-	-	-	-	-	-	-	-	-	-	-	-	18.1<br>(360)	90<br>*<br>(360)
**005-003/ M**	330	ND	8.0	4.7	8.0	71.5<br>*<br>(660)	51<br>*<br>(660)	-	-	-	-	-	-	-	-	-	-	-	-	-	-	7.4<br>(660)	7.0<br>(660)
**002-016/ F**	330	< 3.5	< 6.0	< 3.5	< 6.0	< 3.5<br>(600)	8.0<br>(600)	4.7<br>(870)	7.0<br>(870)	-	-	-	-	-	-	-	-	-	-	-	-	10.5<br>(870)	15<br>*<br>(870)
**003-005/ M**	330	< 3.5	17	5.1	20	3.8<br>(660)	14<br>(660)	-	-	-	-	-	-	-	-	-	-	-	-	-	-	7.1<br>(660)	19<br>(660)
**001-006/ M**	330	< 3.5	21	5.8	24<br>*	3.7<br>(660)	21<br>(660)	-	-	-	-	-	-	-	-	-	-	-	-	-	-	ND	ND
**001-007/ F**	330	< 3.5	< 6.0	< 3.5	< 6.0	< 3.5<br>(660)	< 6.0<br>(660)	-	-	-	-	-	-	-	-	-	-	-	-	-	-	11.9<br>(660)	17<br>*<br>(660)
**001-008/ F**	330	< 3.5	< 6.0	ND	ND	4.7<br>(660)	< 6.0<br>(660)	8.7<br>(990)	7.0<br>(990)	16.4<br>(1320)	17<br>*<br>(1320)	17.6<br>*<br>(1650)	12<br>(1650)	28.5<br>*<br>(1980)	14<br>(1980)	27<br>*<br>(2310)	15<br>*<br>(2310)	20.7<br>*<br>(2640)	12<br>(2640)	16<br>(2970)	16<br>*<br>(2970)	22.2<br>*<br>(2970)	15<br>*<br>(2970)
**003-006/ M**	330	9.7	13	6.6	13	5.6<br>(660)	15<br>(660)	-	-	-	-	-	-	-	-	-	-	-	-	-	-	13.8<br>(660)	23*<br>(660)
**003-007/ M**	330	< 3.5	23<br>*	4.2	12	ND	ND	6.6<br>(990)	11<br>(990)	11.2<br>(1320)	14<br>(1320)	4.7<br>(1650)	15<br>(1650)	7.5<br>(1920)	22<br>(1920)	8.7<br>(2190)	23<br>*<br>(2190)	16.5<br>(2460)	23<br>*<br>(2460)	-	-	7.3<br>(2460)	18<br>(2460)
**004-002/ F**	330	4.9	8.0	5.4	<6.0	7.2<br>(660)	7.0<br>(660)	-	-	-	-	-	-	-	-	-	-	-	-	-	-	14.4<br>(660)	13<br>(660)
**002-018/ F**	330	ND	9.0	9.6	9.0	6.4<br>(660)	< 6.0<br>(660)	-	-	-	-	-	-	-	-	-	-	-	-	-	-	ND	20<br>*<br>(660)
**001-012/ F**	330	ND	ND	4.7	17<br>*	5.2<br>(660)	14<br>(660)	3.6<br>(960)	10<br>(960)	-	-	-	-	-	-	-	-	-	-	-	-	<3.5<br>(960)	8.0<br>(960)
**003-008/ F**	330	< 3.5	<6.0	3.9	<6.0	3.9<br>(660)	<6.0<br>(660)	5.1<br>(990)	<6.0<br>(990)	-	-	-	-	-	-	-	-	-	-	-	-	ND	ND
**003-009/ M**	330	9.6	28<br>*	4.4	41<br>*	-	-	-	-	-	-	-	-	-	-	-	-	-	-	-	-	ND	ND
**002-019/ F**	330	ND	7.0	ND	6.0	4.8<br>(660)	<6.0<br>(660)	-	-	-	-	-	-	-	-	-	-	-	-	-	-	17.8<br>*<br>(660)	16.0<br>*<br>(660)
**005-007/ F**	330	6.0	7.0	7.3	10.0	9.6<br>(600)	11.0<br>(600)	-	-	-	-	-	-	-	-	-	-	-	-	-	-	10.0<br>(600)	17<br>*<br>(600)

CAD = cumulative L-Annamycin for Injection dose at the time of lab draw; F = female; M = male; ND = troponin-T or -I testing not done at ICON due to investigational site error or due to samples being received out of stability; - = not applicable as troponin assessments were not performed because subject did not receive additional cycle

[a] L-Annamycin for Injection administered on Day 1 followed by 20 days off study drug. Additional cycles (if indicated) were administered every 3 weeks.

[b] Troponin testing performed at ICON Central Lab. ICON normal reference range (F): troponin-I ≤ 17.0 ng/L; troponin-T ≤ 14.0 ng/L; ICON normal reference range (M): troponin-I ≤ 35.0 ng/L; troponin-T ≤ 22.0 ng/L; per protocol, blood samples for assessment of troponin-I and -T were collected at Screening, pre-dose at Cycle 1 Day 1 (did not need to be repeated if screening samples were collected within 21 days of Cycle 1 Day 1), on Day 1 of all subsequent cycles of L-Annamycin (within 2 hours after completion of the infusion), and at the End of Treatment (EOT) visit (if not performed within the last 7 days).

* Indicates troponin value outside of normal range. All abnormal results noted for the MB-107 study above were assessed as non-clinically significant by treating physicians.

Overall, with the exception of MB-106 Subject 103-002, all abnormal troponin values were assessed by treating investigators as non-clinically significant. For subjects with abnormal troponin values, review of the available cardiac safety data, including echocardiographic findings, ECG findings where available, and clinical context, did not identify evidence suggestive of L-Annamycin-induced cardiotoxicity. Overall, the available troponin data did not identify a pattern suggestive of L-Annamycin-associated myocardial injury.
